# Cross-cultural adaptation of the Spanish MINICHAL instrument into English for use in the United Kingdom

**DOI:** 10.1186/s12955-022-01943-9

**Published:** 2022-03-04

**Authors:** Andrew N. Jordan, Christine Anning, Lindsay Wilkes, Claire Ball, Nicola Pamphilon, Christopher E. Clark, Nicholas G. Bellenger, Angela C. Shore, Andrew S. P. Sharp, Jose M. Valderas

**Affiliations:** 1 NIHR Exeter Clinical Research Facility, Vascular Medicine, University Hospitals Dorset, Exeter, UK; 2 Institute of Biomedical and Clinical Science, University of Exeter Medical School, Exeter, EX2 5AX UK; 3 Health Services and Policy Research Group, Exeter Collaboration for Academic Primary Care (APEx), NIHR School for Primary Care Research, University of Exeter, Smeall Building, St Luke’s Campus, Magdalen Road, Exeter, EX1 2LU UK; 4 Department of Cardiology, Royal Devon and Exeter Hospital, Exeter, UK

**Keywords:** Hypertension, Adaptation, Validation, MINICHAL

## Abstract

**Background:**

Hypertension is a highly prevalent condition, with optimal treatment to BP targets conferring significant gains in terms of cardiovascular outcomes. Understanding why some patients do not achieve BP targets would be enhanced through greater understanding of their health-related quality of life (HRQoL). However, the only English language disease-specific instruments for measurement of HRQoL in hypertension have not been validated in accordance with accepted standards. It is proposed that the Spanish MINICHAL instrument for the assessment of HRQoL in hypertension could be translated, adapted and validated for use in the United Kingdom. The aim of the study was therefore to complete this process.

**Methods:**

The MINICHAL authors were contacted and the original instrument obtained. This was then translated into English by two independent English-speakers, with these versions then reconciled, before back-translation and subsequent production of a 2nd reconciled version. Thereafter, a final version was produced after cognitive debriefing, for administration and psychometric analysis in the target population of patients living in the Exeter area (Southwest UK) aged 18–80 years with treatment-naïve grade II-III hypertension, before, during and after 18 weeks’ intensive treatment.

**Results:**

The English-language instrument was administered to 30 individuals (median age: 58.5 years, 53% male). Psychometric analysis demonstrated a floor effect, though no ceiling effect. Internal consistency for both state of mind (StM) and somatic manifestations (SM) dimensions of the instrument were acceptable (Cronbach’s alpha = 0.81 and 0.75), as was test–retest reliability (ICC = 0.717 and 0.961) and construct validity, which was measured through co-administration with the EQ-5D-5L and Bulpitt-Fletcher instruments. No significant associations were found between scores and patient characteristics known to affect HRQoL. The EQ-5D-5L instrument found an improvement in HRQoL following treatment, with the StM and SM dimensions of the English language MINICHAL trending to support this (d = 0.32 and 0.02 respectively).

**Conclusions:**

The present study details the successful English translation and validation of the MINICHAL instrument for use in individuals with hypertension. The data reported also supports an improvement in HRQoL with rapid treatment of grade II-III hypertension, a strategy which has been recommended by contemporaneous European guidelines.

*Trial registration* ISRCTN registry number: 57475376 (assigned 25/06/2015).

## Background

Hypertension affects over 1 billion people worldwide, a number which is expected to rise to 1.5 billion by 2025 [1]. The benefits of blood pressure (BP) control are well-established, with a halving of cardiovascular risk for each incremental reduction of 20/10 mmHg down to 115/80 mmHg [2]. Despite these clear benefits of effective antihypertensive therapy, BP control is achieved in only 63% of patients with treated hypertension in England [3].

Non-adherence to medical therapy is an important contributor to apparent treatment-resistant hypertension, as shown by the incorporation of drug level assays and directly-observed therapy into specialist hypertension clinics [4–6]. Poor adherence to medication regimens is particularly understandable for a condition in which the majority of patients are asymptomatic before treatment, considering that diagnosis and treatment have the potential to negatively impact health-related quality of life (HRQoL). It is therefore clear that addressing the HRQoL of patients with hypertension should form part of the holistic approach to care for these individuals, particularly given the association between impaired subjective wellbeing and cardiovascular events [7–9].

Previous studies have shown HRQoL to be reduced in those with a diagnosis of hypertension compared with control subjects [10, 11]. A cross-sectional study has demonstrated that this may, at least in part, be owing to patients’ awareness of their diagnosis [12], with higher perception of well-being found in those who were incidentally hypertensive but not treated, as compared to those with a known diagnosis of hypertension. Additionally, comorbid disease, medication side effects or under-reported symptoms attributable to hypertension (such as mood change, headache or dizziness) may adversely affect HRQoL. Treatment of hypertension, for example with 1 month of angiotensin receptor blockade [13], improves HRQoL in longitudinal studies. BP reduction and achievement of target BP following combination therapy have also been shown to be positive influencers on perceived well-being [14]. Monitoring of HRQoL during treatment may therefore provide a useful tool in determining those participants at higher risk of adverse events or non-adherence.

To date, studies exploring HRQoL in hypertensive subjects have employed generic instruments alone (such as the EuroQol-5D or SF-36) or in combination with disease-specific instruments [10–13]. Disease-specific instruments are valued as they are felt to be more responsive to change; it is unlikely that generic instruments are able to adequately capture HRQoL in all populations suffering from all types of conditions [15]. Although disease-specific instruments for hypertension have been validated in Spanish [16, 17], Brazilian Portuguese [18] and Italian [19], those in English, such as the Bulpitt-Fletcher questionnaire [20], have not undergone appropriate validation according to current standards [21, 22] (Table 1).

**Table 1:** Disease-specific instruments for the evaluation of HRQoL in hypertension

Instrument	Original language	Number of items	Year of first publication	Previous cross-cultural adaptation	Modern validation
Bulpitt-Fletcher	English	46	1990	Dutch, Portuguese (Brazil)	No
MINICHAL	Spanish	17	2002	Portuguese (Brazil)	Yes
CHAL	Spanish	55	2000	None	Yes
HYPER31	Italian	35	1995	Arabic (Egypt), German, Russian	Yes

Considering the Bulpitt-Fletcher instrument in detail, a degree of redundancy can be demonstrated, with 11 of 46 questions not contributing to the overall score as per the scoring methods proposed by the authors [20]. The Bulpitt-Fletcher instrument has a not trivial administrative burden, with an estimated administration time of 20–40 min [20], which is incongruent with the notion that questionnaires should be kept short and simple to minimize measurement error [23] and further limiting its widespread adoption. In addition, not all questions within the Bulpitt-Fletcher instrument can be applied to all participants, with question 35 applying to men only and question 37 to only those in paid employment. This will inevitably lead to missing data when the instrument is administered, impacting on its performance. Further, the Bulpitt-Fletcher instrument includes several items (items 11, 14, 32 and 37) that place it overall among clinimetric measures rather than psychometric measures and hence better placed to estimate disease severity than measuring HRQoL [24, 25]. Finally, given that both treatment and general patient perceptions and expectations have changed markedly since the inception of the Bulpitt-Fletcher instrument, it may no longer capture the somatic manifestations of side effects from first-line medications or up-to-date cultural values affecting HRQoL. For example, questions 21, 25 and 35 aim to establish common side effects of beta-blockers, which are no longer considered a mainstay of treatment for hypertension [26] and questions related to sexual activity (questions 31–35) may raise more doubts regarding the safe storage of sensitive personal data in the digital age.

**Fig. 1:**
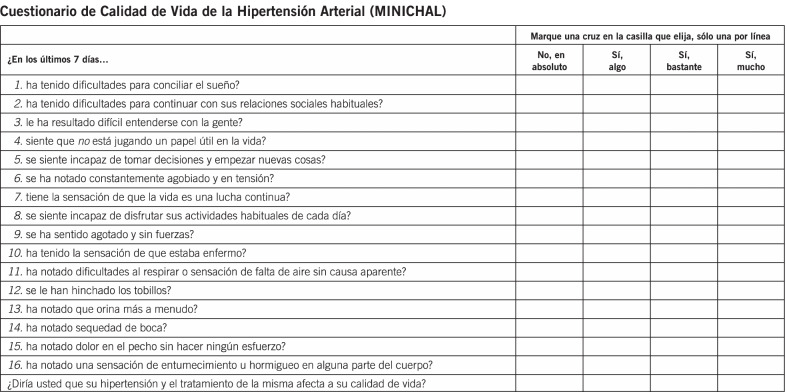
Original MINICHAL instrument

Adapting and validating the readily available Spanish MINICHAL hypertension disease-specific instrument in English offers therefore obvious advantages and may be an efficient alternative to the development of a new instrument. The MINICHAL instrument, originally conceived and validated in Spanish [17] (Fig. 1), has an average administration time of just over 7 min. Although 17 questions are described, the final question pertains to the subject’s overall perception of their own health; it is not included in the scoring (or the validation) of the original instrument. Within the remaining 16 questions, 2 domains have been determined: State of Mind (StM) and Somatic Manifestations (SM). Scores range from 0 to 30 for StM and from 0 to 18 for SM; lower scores reflect higher HRQoL [17]. Psychometric evaluation [16, 17] has shown that it meets current standards for internal consistency, test–retest reliability and responsiveness to change. Validation of the MINICHAL instrument has been confirmed through co-administration with 2 generic instruments and responsiveness to change evaluated following 6 months of antihypertensive treatment intensification, finding a positive correlation between degree of BP (and heart rate) reduction and improvement in the MINICHAL score, especially the StM domain [16].

The aim of this study was therefore to translate, adapt and evaluate the psychometric performance of the existing MINICHAL for its use in the United Kingdom. Within this, we aimed to test responsiveness to change of the instrument through administration before and after an 18-week intensive treatment programme for subjects with newly diagnosed grade II-III hypertension [27].

## Methodology

### Study design, setting and sample size

The study consisted of two components: the adaptation of the MINICHAL instrument into English following the guidelines set out by the International Society for Quality of Life Research (ISOQOL) [28], International Society for Pharmacoeconomics and Outcomes Research (ISPOR) [22] and the Evaluating the Measurement of Patient-Reported Outcomes (EMPRO) tool [21], and the further evaluation of the instrument within the setting of a clinical study for the treatment of hypertension.

An open label single-centre cohort study was employed, using participants enrolled in an 18-week treatment programme for newly-diagnosed grade II-III hypertension [27]. This treatment programme aimed to enroll 50 participants and it was envisaged that the new instrument be applied to all participants enrolled subsequent to completion of the necessary translation and cross-cultural steps.

### Participants

Participants were recruited from 22 primary care practices or from secondary care in the county of Devon, United Kingdom. Referred subjects were eligible for screening if they were aged 18–79 years, had an office systolic BP of ≥ 170 mmHg and had never previously received antihypertensive treatment.

Exclusion criteria were: Glomerular Filtration Rate (GFR) < 60 ml/min/1.73m^2^, previous renal artery intervention, haemoglobin < 10 g/dl, platelet count < 100 × 10^9^/l, bleeding diathesis, pregnancy or breastfeeding, inability to provide informed consent, hypertension-related event (including stroke or acute kidney injury) within the preceding 3 months, or any condition, including hypertensive urgency, requiring more immediate BP lowering or tailored antihypertensive strategy at enrolment.

At screening, subjects underwent 24-h ambulatory BP monitoring (ABPM) and were eligible for trial participation if this confirmed at least grade 2 hypertension with a daytime average systolic BP (DASBP) measurement ≥ 150 mmHg.

All participants gave informed consent and followed a treatment protocol using an antihypertensive medication pathway with appointments every 2–4 weeks over an 18-week period [27].

### Issues of interest (exposure)

1. Translation and cross-cultural adaptation

Firstly, the MINICHAL instrument was obtained from its original publication in Spanish [16] (Fig. 1); and permission for adaptation secured through contact with authors of the original publication.

Forward translation was provided by 2 independent native English-speakers. This version was discussed by a panel of researchers with experience of cross-cultural adaptation, including 1 of the aforementioned translators, producing a consensus for each of the 16 questions in turn. This process produced the first reconciled version of the English instrument.

The first reconciled version was then back-translated by a third independent native Spanish-speaking professional translator. To ensure that language equivalence between questionnaires had been achieved, this version was compared with the original Spanish MINICHAL to highlight discrepancies, allowing the panel to produce a second reconciled version of the questionnaire.

Following this, the second reconciled version underwent cognitive debriefing (pilot testing using the techniques “thinking aloud”, probing and debriefing). Harmonization with previous Brazilian Portuguese translation of the instrument was also completed at this stage [18]. The results informed the production of the final version of the adapted instrument.

2. Evaluation of psychometric properties

Accepted standards for psychometric evaluation of the instrument were followed as per ISOQOL [28], ISPOR [22] and EMPRO [21]. The metric qualities were determined through administration of the MINICHAL instrument (together with the EQ-5D-5L and Bulpitt-Fletcher instruments) at weeks 0, 8, 10 and 18 of the treatment programme. The instruments were self-administered, being given to participants at the time of their study appointments and returned to the study team by post once completed. The order of instruments within the survey was: Bulpitt-Fletcher, then EQ-5D-5L, followed by the MINICHAL instrument. Returned data was collected and stored by the study team in a protected electronic database in accordance with local protocols and NIHR Good Clinical Practice.

As in the original validation and subsequent cross-cultural adaptation, the StM and SM domains were reported and the final question of the instrument reflecting the subjects overall assessment of their HRQoL was not used in the analysis [16–18].

### Comparison

The English-language MINICHAL instrument responses were evaluated for internal consistency and construct validity. Test–retest reliability was evaluated through administration of the instrument twice at weeks 8 and 10 after enrollment, between which times no change in medication occurred.

Construct validity was also evaluated through co-administration with a generic instrument (EQ-5D-5L) and hypertension-specific instrument (Bulpitt-Fletcher instrument).

The EQ-5D-5L instrument is a widely available generic measure of healthcare status [29] consisting of two components: the visual analogue scale (VAS) and index score. The VAS assesses HRQoL between 0 (worst imaginable health) and 100 (best imaginable health), with the participant marking this on a 0–100 scale and writing their self-determined numerical score within a specified box on the same page. The index score requests that the participant generate a response (within 5 levels) to 5 items, comprising the dimensions of mobility, self-care, usual activities, pain/discomfort and anxiety/depression [30, 31]. These responses are then converted into a single score using country-specific weighting and conversion tables, with higher scores indicating greater HRQoL. As such, the EQ-5D-5L exhibits excellent psychometric properties across a wide range of conditions [32].

The Bulpitt-Fletcher instrument was developed as a disease-specific instrument for the evaluation of HRQoL in hypertension more than 30 years ago [20]. 46 self-administered questions are included, with some requiring descriptive responses. A scoring index is produced as a consequence of the answers received, ranging from 0 (death) to 1 (perfect health), though the instrument has not be validated according to modern standards.

Included within the validation process was the administration of all instruments before and after completion of the 18-week treatment programme, with this forming the basis of the assessment of the new English-language MINICHAL instrument’s responsiveness to change.

### Ethics and endpoint

The primary purpose of the study was the translation and validation of the Spanish MINICHAL instrument into English for use in the United Kingdom. The secondary endpoint of the study was the effect on HRQoL of an intensive 18-week antihypertensive treatment programme for individuals with treatment-naïve grade II-III hypertension.

Ethical approvement for this study was agreed prospectively (NRES Committee South West ref. 15/SW0077). All participants gave voluntary informed consent in accordance with the Declaration of Helsinki.

### Statistical analysis

Baseline and outcome data are presented as means (standard deviation) or medians (interquartile range) for continuous data depending on the normality of the data and counts (percentages with 95% confidence intervals) for categorical and binary variables. Parametric data were analyzed using a paired t-test; non-parametric data were analyzed using Wilcoxon’s signed ranks test.

Internal consistency was determined by calculating pairwise correlations between items and Cronbach’s alpha. Test–retest reliability was evaluated using the intraclass correlation coefficient. Given the reliability of 0.80 in the original MINICHAL instrument evaluation [17], a 2-tailed intraclass correlation coefficient was expected to be > 0.70 (α = 0.05).

Construct validity was assessed through co-administration with a generic questionnaire (EQ-5D-5L) and the hypertension-specific Bulpitt-Fletcher instrument (Spearman correlation), predicting a moderate to low correlation with the EQ-5D-5L instrument and moderate to high correlation with the Bulpitt-Fletcher instrument. Construct validity was further evaluated through association with variables known to affect quality of life measures in hypertensive patients, including heart rate, body mass index (BMI), number of antihypertensive agent, BP and female gender [13, 33] (Mann–Whitney test and Pearson’s correlation coefficient). As reported in the original evaluation of the MINICHAL instrument, it was predicted that female gender would be associated with an increased score in the StM domain, increased age and increased BMI would be associated with an increased score in the SM domain and raised BP and number of co-morbidities would correlate with an increased score in both domains [17].

Responsiveness to change was evaluated through comparison of the MINICHAL scores before and after 18 weeks’ intensive antihypertensive treatment (paired t test, following test of normality, and effect size). It was anticipated that this intervention would affect HRQoL as a previous meta-analysis has found a small but significant improvement in general well-being following active treatment of hypertension (d = 0.139) [34], including whilst using the same pharmacological groups employed in the treatment protocol, with the potential for accelerated treatment in our protocol to accentuate HRQoL gains.

In the original evaluation of the Spanish MINICHAL instrument, re-testing 6 months after treatment intensification was found to improve HRQoL, with an effect size of 0.55 and 0.46 for the StM and SM domains respectively. Moreover, there was a significant correlation between improvement in HRQoL and BP reduction [17]. In the present study, univariate linear regression models were used to determine the relationship between BP response during the study and instrument scores as outcome variables, with the hypothesis that change in HRQoL would be related to degree of BP reduction, particularly the StM domain of the MINICHAL instrument, as previously reported.

A two-sided *P* value threshold < 0.05 was considered statistically significant. Statistical analysis was performed using STATA v16.0 (StataCorp, College Station, Texas, USA).

## Results

### Translation

Differences between the forward translations were noted, in particular the grading of responses to each question being translated as “not at all, yes occasionally, yes quite often and yes often” compared with “no not at all, yes a little, yes a fair amount and yes a lot”. Reconciliation was achieved through a meeting between the investigators and one of the forward translators, with phraseology chosen to provide a more distinct gradient of response in the final reconciled version: “no not at all, yes a little, yes a moderate amount and yes a lot”. In addition, the two forward translations for question 3 were markedly different, focusing on “being understood by people” versus “getting on with other people”, requiring reconciliation to “Have you had difficulties communicating with other people?”, in order to combine both interpretations of the question. Question 8 produced differing translations: “everyday activities” versus “normal routine”, with “usual activities” chosen for the reconciled instrument as this reflected the senses of both translations. For question 11, “shortness of breath” was felt to be easier to understand than the alternative translation of “feeling of suffocation”. In question 15, “exertion” was selected over “straining”, as it was concluded that the authors of the instrument were seeking to elicit a symptom of ischaemic heart disease rather than muscular chest wall pain on straining.

The reconciled version of the instrument was then back-translated by a native Spanish speaker. This highlighted a difference in question 15 compared with the original Spanish version, with “without having exerted yourself” rephrased to “without physical exertion” as a consequence. The overall grading of response also differed in the Brazilian Portuguese translation when compared with the English back translation, though it was felt that the Portuguese translation lacked sufficient gradient of response between “yes a lot” and “yes very much” and therefore the second reconciled version was preferred.

**Fig. 2:**
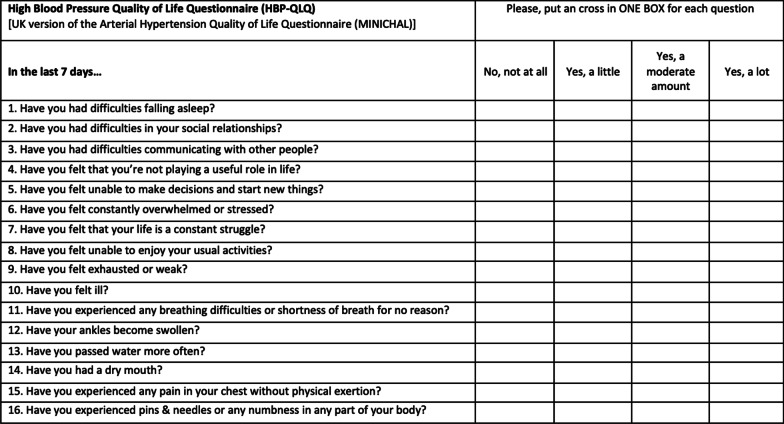
English-language MINICHAL instrument

Cognitive debriefing took place with 8 individuals with typical demographics for the target population: the median age was 60 years, 50% were educated to undergraduate degree level or higher and 50% were female. All participants in the cognitive debriefing process reported no difficulties in understanding the questions as drafted in the second reconciled version. No consistent changes to the instrument were suggested and, in light of these findings, a final version of the instrument was approved for evaluation (Fig. 2).

### Instrument evaluation

The final version of the instrument was administered to 30 native English speakers before and after antihypertensive treatment. Of these, 53% were male, median age was 58.5 years and mean pre-treatment office BP measured 171/101 mmHg, falling to 130/80 mmHg after 18 weeks of treatment. The characteristics of these participants are given in Table 2.

**Table 2:** Participant characteristics

Variable	Before intervention	After intervention	P value
Office systolic BP (mmHg)	171 ± 15.8	130 ± 10.6	< 0.0001
Office diastolic BP (mmHg)	101 ± 11.5	80 ± 8.7	< 0.0001
Daytime average systolic BP (mmHg)	164 ± 12.2	134 ± 10.8	< 0.0001
Daytime average diastolic BP (mmHg)	93 ± 10.1	78 ± 6.8	< 0.0001
Heart rate (bpm)	69 ± 10.9	66 ± 9.6	0.024
BMI (kg/m^2^)	30.0 ± 5.9	29.9 ± 5.5	0.79
Current smoker (n)	2 (7%)	2 (7%)	1.00^^^
Alcohol (units/week)	7 (1–15)	2 (1–10)	0.36^§^
Angiotensin receptor blocker (n)	0	25 (83%)	n/a
Calcium channel blocker (n)	0	29 (97%)	n/a
Thiazide diuretic (n)	0	15 (50%)	n/a
Aldosterone antagonist (n)	0	4 (13%)	n/a
α-blocker (n)	0	1 (3%)	n/a
β-blocker (n)	0	1 (3%)	n/a
Number of anti-hypertensives (n)	0	2.5 (2–3)	n/a
Number of other medications (n)	0 (0–1)	0 (0–1)	0.32^§^
Number of co-morbidities (n)	1.0 ± 0.9	1.0 ± 0.9	1.00

Expressed as mean ± standard deviation or median and interquartile range

^§^Wilcoxon’s signed ranks test

^^^One-sample test of proportions

#### Floor and ceiling effects

For each item, the minimum score was returned in 67–97% responses, with the greatest floor effect seen in item 3: “Have you had difficulties communicating with other people?”. The maximum score for an item was returned in 0–3% responses, indicating no ceiling effect, as previously reported for the Spanish version of the instrument [17].

#### Reliability

Reliability (internal consistency) was acceptable for both the StM and SM domains: Cronbach’s alpha 0.81 and 0.75 respectively. As 1 participant did not return completed questionnaires for the evaluation of test–retest reliability, the intraclass correlation coefficient (ICC) between the scores derived for the remaining 29 participants who underwent test–retest data acquisition between weeks 8 and 10 of treatment was calculated, with no change in medication undertaken between these two appointments. This determined acceptable test–retest reliability for both domains: StM ICC = 0.717 (95% CI 0.378–0.913); SM ICC = 0.961 (95% CI 0.876–0.988).

#### Validity

Both StM and SM domains significantly correlated with the EQ-5D-5L index score, EQ-5D-5L VAS and the Bulpitt-Fletcher instrument (Table 3). Correlations between the MINCHAL domains and all non-descriptive Bulpitt-Fletcher questions without a high degree of redundancy are provided in “Appendix”.

**Table 3:** Correlations between MINICHAL domains and other HRQoL measurements

Instrument/dimension	StM		SM	
	r_s_	P	r_s_	P
EQ-5D-5L VAS	− 0.394	0.0019	− 0.362	0.0045
EQ-5D-5L index score (UK)	− 0.500	< 0.0001	− 0.491	0.0001
EQ-5D-5L mobility dimension	0.3105	0.0157	0.3929	0.0019
EQ-5D-5L self-care dimension	0.1127	0.3913	0.2237	0.0858
EQ-5D-5L usual activities dimension	0.5068	< 0.0001	0.5871	< 0.0001
EQ-5D-5L pain/discomfort dimension	0.4258	0.0007	0.4659	0.0002
EQ-5D-5L anxiety/depression dimension	0.5382	< 0.0001	0.3267	0.0108
Bulpitt-Fletcher (overall index)	− 0.472	0.0001	− 0.291	0.0243
Bulpitt-Fletcher Q1 (lightheadedness)	0.1687	0.195	0.1690	0.1969
Bulpitt-Fletcher Q4 (daytime somnolence)	0.4663	0.0002	0.3111	0.0156
Bulpitt-Fletcher Q8 (breathlessness)	0.3591	0.0052	0.3997	0.0017
Bulpitt-Fletcher Q9 (ankle swelling)	0.1502	0.2562	0.3698	0.0039
Bulpitt-Fletcher Q27 (headache)	0.5489	< 0.0001	0.2257	0.0829
Bulpitt-Fletcher Q39 (impaired usual activities)	0.4191	0.0009	0.2943	0.0225

Spearman’s correlation coefficient (r_s_)

StM: State of Mind, SM: Somatic Manifestations, VAS: Visual Analogue Scale

**Fig. 3:**
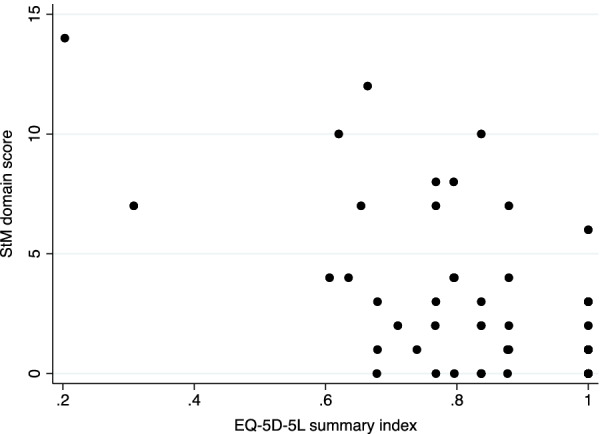
Correlation between the MINICHAL StM domain and EQ-5D-5L summary index. *R*_*s*_ = − 0.500, *p* < 0.0001

All correlations were moderate, including a correlation found between the StM domain and the EQ-5D-5L index score (UK values) (Fig. 3). The StM domain showed a higher correlation with the Bulpitt-Fletcher questionnaire than the SM domain. Within the same instruments, correlations were determined between the StM and SM dimensions of the English MINICHAL (r_s_ = 0.5257; p < 0.0001) and between the EQ-5D-5L index score and EQ-5D-5L VAS (r_s_ = 0.3725; p = 0.0034).

No significant difference was found between genders for either the StM domain (female: 2 (1–5); male: 2 (1–4.5); p = 0.164) or SM domain (female: 1 (0–2); male: 1 (0–3); p = 0.901). Pearson’s correlation was used to explore relationships between scores and patient characteristics. Although most of the associations were in the direction predicted by our hypotheses, none of them were found to be statistically significant (Table 4).

**Table 4:** Correlations between MINICHAL scores and subject demographic and clinical characteristics

Variable	StM		SM	
	r	P	r	P
Age	− 0.068	0.608	0.128	0.331
BMI (kg/m^2^)	0.024	0.858	0.105	0.426
Office sBP	− 0.044	0.737	− 0.004	0.975
Office dBP	− 0.034	0.796	− 0.181	0.166
Daytime average sBP	0.079	0.551	0.034	0.798
Daytime average dBP	− 0.001	0.993	− 0.187	0.152
Heart rate	− 0.117	0.372	− 0.194	0.138
No. of antihypertensive medications	− 0.198	0.129	− 0.025	0.851

Pearson’s correlation coefficient (r)

StM: State of Mind, SM: Somatic Manifestations, sBP: systolic blood pressure, dBP: diastolic blood pressure

#### Responsiveness

Results from the application of the patient-reported quality of life instruments before and after 18 weeks of intensive antihypertensive treatment are summarized in Table 5.

**Table 5:** Change in patient-reported quality of life after 18 weeks’ intensive antihypertensive treatment

	Pre-treatment score	Week 18 score	P^**§**^	Effect size (d)
EQ-5D-5L VAS	79.6 ± 13.54	88.8 ± 8.11	0.0001	0.82
EQ-5D-5L index score (UK)	0.84 ± 0.18	0.87 ± 0.17	0.05	0.17
Bulpitt-Fletcher	0.88 ± 0.017	0.91 ± 0.017	0.049	1.76
MINICHAL StM	3.23 ± 3.52	2.17 ± 3.03	0.076	0.32
MINICHAL SM	1.33 ± 1.86	1.30 ± 2.11	0.932	0.02

Expressed as mean ± standard deviation

StM: State of Mind, SM: Somatic Manifestations, VAS: Visual Analogue Scale

d: Cohen’s

^§^Paired t-test

Following 18 weeks of intensive hypertension treatment there was a significant improvement in quality of life as measured by the EQ-5D-5L, in particular the VAS (d = 0.82). Application of the MINICHAL instrument produced results in agreement with this, with a greater responsiveness of the StM domain (d = 0.32) when compared with the SM domain (d = 0.02), though these did not reach statistical significance. On the contrary, the Bulpitt-Fletcher instrument found a significant reduction in HRQoL following treatment.

Office systolic BP response to treatment was not associated with a change in any of the measures of HRQoL (StM domain (p = 0.342), SM domain (p = 0.406), EQ-5D-5L VAS (p = 0.532), EQ-5D-5L index score (p = 0.740) or Bulpitt-Fletcher scores (p = 0.553)). Neither was the case for daytime average systolic BP measured with ambulatory BP monitoring (data not shown).

## Discussion

We report the first validation of a disease-specific English-language patient-reported outcome instrument for use in hypertension. The successful translation and validation of the instrument was completed in accordance with accepted standards [21, 22, 28].

Evaluation of the instrument demonstrated an important floor effect though no ceiling effect. This is in-keeping with the initial evaluation of the Spanish MINICHAL instrument [17] and reflects a relatively low symptom burden for the majority of subjects with hypertension. Internal consistency for the English MINICHAL instrument comfortably met current standards for use at group level, with dimension analysis for StM and SM finding similar values to those reported for the Spanish iteration of the instrument (0.81 and 0.75 vs 0.87 and 0.75 respectively) [17]. A similar situation was observed for test-rest reliability and, in this case, the English version of the instrument also compared favorably with the Spanish instrument.

Construct validity was confirmed through the instrument’s correlation with generic instruments (EQ-5D-5L index score and EQ-5D-5L VAS) and the disease-specific Bulpitt-Fletcher instrument. In terms of strength of association, this was greatest with the EQ-5D-5L index score and weakest with the Bulpitt-Fletcher instrument. This is not completely surprising considering that the Bulpitt-Fletcher instrument, though developed specifically for hypertension, is not an ideal instrument for measuring quality of life because of its mixed clinimetric-psychometric approach. A higher correlation between two instruments whose main focus is HRQoL can be therefore expected [31].

Responsiveness was tested through administration of the instrument before and after 18 weeks’ intensive treatment of hypertension, using medications and medication combinations recommended in current international guidelines, though over an accelerated timeframe [27], an intervention that seemed to have a measurable significant impact on generic quality of life as measured through the VAS of the EQ-5D-5L (large effect size), but not on the EQ-5D-5L index score (small effect size). The effect size for the StM scale was larger than for the EQ-5D-5L index score, but not statistically significant due to a high dispersion of scores, and was negligible for the SM scale. It must be noted that scores were already very low for the latter scale, suggesting that the floor effect observed in this group of patients may have limited our ability to detect improvement.

A meta-analysis of cross-sectional studies has found that HRQoL is impaired across all eight domains of the SF-36 and SF-12 instruments in those with hypertension, when compared with normotensive individuals [35]. Subsequent investigation has found reduced HRQoL in patients treated for hypertension, when compared with untreated hypertensive subjects [36], which may be related in large part to the subjects’ awareness of their diagnosis [37]. However, these conclusions are limited by the inherent bias imparted by the cross-sectional nature of their design. In terms of longitudinal studies, which enable subjects to act as their own control group thereby minimizing confounding factors, improvement in HRQoL following treatment of hypertension has been demonstrated in a meta-analysis [34]. This observation can be found with a variety of antihypertensive agents [38]. Our report of improved HRQoL following treatment is therefore in-keeping with previous longitudinal data. Moreover, given that visits were predominantly delivered by allied healthcare professionals within the clinical study, our finding mirrors that of a recent Cochrane review, which concluded that HRQoL, in particular the physical functioning domain, improves with treatment of hypertension delivered by allied healthcare professionals [39]. This would be also in line with the lack of responsiveness observed in this study for the SM scale.

In addition, our intervention was delivered in an accelerated timeframe, a manner of treatment delivery which is known to improve HRQoL in other fields of medicine, such as hip arthroplasty [40] and radiotherapy in the treatment of breast cancer [41]. It would therefore be reasonable to propose that the improvement in HRQoL conferred through treatment of hypertension will have been accentuated by the rapid treatment protocol employed in this study.

Conversely, administration of the Bulpitt-Fletcher instrument within the protocol found a significant reduction in HRQoL following hypertension treatment. Notably, psychometric evaluation with measurement of internal consistency, floor effect, ceiling effect and construct validity for the instrument has not been reported. Additionally, test–retest reliability has only been examined for selected concepts within the questionnaire, rather than the instrument itself [42]. However, responsiveness to change of the Bulpitt-Fletcher questionnaire has previously been reported through administration of the instrument within clinical studies, such as a trial comparing hypertension treatment with verapamil versus propranolol [43] and a further study comparing captopril with atenolol [44]. Although generic instruments were co-administered with the Bulpitt-Fletcher instrument within these studies, no direct statistical comparison was conducted and therefore construct validity was not evaluated.

Dimensions analysis of the MINICHAL instrument results revealed a nominally greater responsiveness within the StM dimension compared to the SM dimension. Different weighting between the EQ-5D-5L and the Bulpitt-Fletcher instruments in terms of somatic symptoms versus psychological well-being may also therefore explain the discrepant results between these two instruments when applied to subjects within the study.

### Future implications

The availability of an English language, short, validated, disease-specific instrument for the evaluation or HRQoL in hypertension is of value, particularly given the high prevalence of this condition and therefore its wide applicability to patients. Non-adherence to treatment is a crucial element of apparent treatment-resistance in hypertension [45] and therefore the ability to monitor the impact of hypertension and its treatment for patients could help address this important limiter to successful treatment. Furthermore, it is anticipated that the newly-adapted and validated English language instrument will be used in future research practice to ensure that new treatment strategies for hypertension positively impact HRQoL.

### Study limitations

The conclusions drawn from the study are limited by the relatively low number of subjects enrolled, a drawback which could be addressed through further deployment of the translated MINICHAL instrument in future studies of hypertension treatment. Furthermore, as this was a before-and-after study, the effects of time, rather than treatment, on HRQoL cannot be discounted from the analysis, though this is limited by the relatively short 18-week treatment phase.

In addition, it is acknowledged that our study cohort was geographically limited to south-west England. Nevertheless, region-specific language is not used within the translated instrument and no difficulties with comprehension or cultural applicability are envisaged should the instrument be used across the United Kingdom.

The evaluation of the MINICHAL instrument alongside the EQ-5D-5L VAS has demonstrated the latter, generic instrument to be more responsive to change than our disease-specific instrument, the converse to the expected. The reason for this is unclear, though may relate to the greater range of responses afforded by the EQ-5D-5L VAS in comparison with the MINICHAL’s 4 response options. Analysis of the components of the EQ-5D-5L index score which changed most during treatment determined that the pain/discomfort and anxiety/depression items returned differing scores most frequently. Although anxiety/depression is covered well by the MINICHAL questions, only one question pertains to pain (“Have you experienced any pain in your chest without physical exertion?”). Therefore, a relative deficiency of the MINCHAL instrument in exploring this aspect of symptoms, together with the limited sample size in the present study, may in part explain this discrepancy.

In light of these findings, we recommend that future studies of hypertension should consider using both the MINICHAL instrument and EQ-5D-5L in tandem for the assessment of HRQoL.

## Conclusions

The study describes the first validation of an English-language disease-specific instrument for use in the assessment of HRQoL in subjects with hypertension. Furthermore, evidence of acceptability for patients in the rapid treatment of moderate and severe hypertension is reported, a treatment strategy which is recommended in the most recent European guidelines [26], though previously without evidence of acceptability for patients.

## Data Availability

The datasets used and/or analyzed during the current study are available from the corresponding author on reasonable request.

## Appendix: Correlations between MINICHAL domains and Bulpitt-Fletcher questions

**Table:**

Bulpitt-Fletcher instrument item	StM		SM	
	r_s_	P	r_s_	P
Overall index	− 0.472	0.0001	− 0.291	0.0243
Q1 (lightheadedness)	0.1687	0.1975	0.1690	0.1969
Q4 (daytime somnolence)	0.4663	0.0002	0.3111	0.0156
Q5 (hours of sleep)	− 0.0218	0.8710	0.0869	0.5165
Q6 (limb weakness)	0.2026	0.1271	0.2274	0.0860
Q7 (visual blurring)	0.1342	0.3198	0.2856	0.0313
Q8 (breathlessness)	0.3591	0.0052	0.3997	0.0017
Q9 (ankle swelling)	0.1502	0.2562	0.3698	0.0039
Q11 (bowel opening/day)	0.0750	0.5723	0.2718	0.0373
Q12 (loose motions)	0.0462	0.7257	− 0.0687	0.6019
Q13 (constipation)	0.3237	0.0116	0.2793	0.0307
Q14 (nocturia)	0.1345	0.3057	0.1206	0.3587
Q15 (dry mouth)	0.2715	0.0358	0.4006	0.0015
Q17 (bad taste in mouth)	0.2580	0.0527	0.3747	0.0041
Q18 (blocked or runny nose)	0.3821	0.0031	0.4472	0.0004
Q20 (facial/neck flushing)	0.1056	0.4220	0.0688	0.6016
Q21 (vivid dreams/nightmares)	0.2518	0.0523	0.2412	0.0634
Q22 (nausea)	0.3919	0.0020	0.3522	0.0058
Q23 (rash)	0.0213	0.8715	0.2076	0.1114
Q24 (pruritis)	0.0448	0.7338	0.1403	0.2848
Q25 (finger discolouration)	− 0.0283	0.8298	− 0.0283	0.8298
Q27 (headache)	0.5489	< 0.0001	0.2257	0.0829
Q30 (dry cough)	0.2033	0.1226	0.1121	0.3978
Q39 (usual activities)	0.4191	0.0009	0.2943	0.0225
Q42 (hobbies)	− 0.0442	0.7373	− 0.1512	0.2185
Q46 (other aspects of life)	0.4300	0.0008	0.2788	0.0357

Spearman’s correlation coefficient (r_s_)

All Bulpitt-Fletcher instrument questions, excluding those which are descriptive or have a large degree of redundancy

## Abbreviations

BMI: Body mass index
BP: Blood pressure
dBP: Diastolic blood pressure
EMPRO: Evaluating the measurement of patient-reported outcomes
HRQoL: Health-related quality of life
ISOQOL: International Society of Quality of Life Research
ISPOR: International Society for Pharmacoeconomics and Outcomes Research
sBP: Systolic blood pressure
SM: Somatic manifestations
StM: State of mind
VAS: Visual analogue scale
